# Effect of ABO blood group on asymptomatic, uncomplicated and placental *Plasmodium falciparum* infection: systematic review and meta-analysis

**DOI:** 10.1186/s12879-019-3730-z

**Published:** 2019-01-25

**Authors:** Abraham Degarege, Merhawi T. Gebrezgi, Consuelo M. Beck-Sague, Mats Wahlgren, Luiz Carlos de Mattos, Purnima Madhivanan

**Affiliations:** 1 Department of Epidemiology, Robert Stempel College of Public Health & Social Work, Florida International University, 11200 SW 8th Street, Miami, FL 33199 USA; 2 Aklilu Lemma Institute of Pathobiology, Addis Ababa University, Addis Ababa, Ethiopia; 3 Department of Health Promotion and Disease Prevention, Robert Stempel College of Public Health & Social Work, Florida International University, Miami, Florida USA; 4 Department of Microbiology, Tumor and Cell Biology (MTC), Karolinska Institute, Stockholm, Sweden; 5 Molecular Biology Department, Faculdade de Medicina de São José do Rio Preto, São José do Rio Preto, SP Brazil; 6 Public Health Research Institute of India, Mysore, India

**Keywords:** ABO blood type, Asymptomatic malaria, Uncomplicated malaria, Placental malaria

## Abstract

**Background:**

Malaria clinical outcomes vary by erythrocyte characteristics, including ABO blood group, but the effect of ABO blood group on asymptomatic, uncomplicated and placental *Plasmodium falciparum* (*P. falciparum*) infection remains unclear. We explored effects of ABO blood group on asymptomatic, uncomplicated and placental *falciparum* infection in the published literature.

**Methods:**

A systematic review and meta-analysis was performed using the preferred reporting items for systematic reviews and meta-analyses guidelines. Articles in Pubmed, Embase, Web of Science, CINAHL and Cochrane Library published before February 04, 2017 were searched without restriction. Studies were included if they reported *P. falciparum* infection incidence or prevalence, stratified by ABO blood group.

**Results:**

Of 1923 articles obtained from the five databases (Embase = 728, PubMed = 620, Web of Science = 549, CINAHL = 14, Cochrane Library = 12), 42 met criteria for systematic review and 37 for meta-analysis. Most studies (*n* = 30) were cross-sectional, seven were prospective cohort, and five were case-control studies. Meta-analysis showed similar odds of uncomplicated *P. falciparum* infection among individuals with blood group A (summary odds ratio [OR] 0.96, 15 studies), B (OR 0.89, 15 studies), AB (OR 0.85, 10 studies) and non-O (OR 0.95, 17 studies) as compared to those with blood group O. Meta-analysis of four cohort studies also showed similar risk of uncomplicated *P. falciparum* infection among individuals with blood group non-O and those with blood group O (summary relative risk [RR] 1.03). Meta-analysis of six studies showed similar odds of asymptomatic *P. falciparum* infection among individuals with blood group A (OR 1.05), B (OR 1.03), AB (OR 1.23), and non-O (OR 1.07) when compared to those with blood group O. However, odds of active placental *P. falciparum* infection was significantly lower in primiparous women with non-O blood groups (OR 0.46, 95% confidence interval [CI] 0.23 – 0.69, I^2^ 0.0%, three studies), particularly in those with blood group A (OR 0.41, 95% CI 0.003 – 0.82, I^2^ 1.4%, four studies) than those with blood group O.

**Conclusions:**

This study suggests that ABO blood group may not affect susceptibility to asymptomatic and/or uncomplicated *P. falciparum* infection. However, blood group O primiparous women appear to be more susceptible to active placental *P. falciparum* infection.

**Electronic supplementary material:**

The online version of this article (10.1186/s12879-019-3730-z) contains supplementary material, which is available to authorized users.

## Background

Malaria caused due to *Plasmodium falciparum* infection remains a major cause of death in tropical and subtropical countries [1, 2]. Individuals infected with *P*. *falciparum* may present with mild (e.g. fever, chills, headaches, nausea, malaise) or severe clinical symptoms (e.g. pulmonary edema, cerebral malaria, acute renal failure, severe anemia) [3, 4]. In malaria endemic areas, some individuals may have *P. falciparum* parasitemia, but not show symptoms suggestive of *Plasmodium* infection (asymptomatic parasitemia) [5, 6]. While severe *P. falciparum* infection may cause diverse organ dysfunction, uncomplicated *P. falciparum* infection usually causes mild health problems such as anemia and undernutrition [3, 4]. On the other hand, asymptomatic *P. falciparum* infection may not cause any significant health problems, although the parasite can persist in the blood for several months and produce gametocytes that can serve as a source of infection for the vector [5, 6]. Thus, asymptomatic infection contributes to the maintenance of malaria transmission in endemic regions [5, 6].

Various genetic variants or red blood cell polymorphisms have been identified that can make humans relatively more susceptible or resistant to *P*. *falciparum* infection and affect clinical outcomes of the disease [7, 8]. One of the genetic factor hypothesized to influence human susceptibility to clinical outcomes of malaria is ABO blood group [7–10]. Many studies have investigated the nature of interaction between ABO blood group and *P. falciparum* infection for decades (reviewed in [9, 10]). A recent meta-analysis study confirmed an increased severity of *P. falciparum* infection among individuals with blood group A, B and AB in comparison with those of blood group O [11]. However, the effect of ABO blood group on asymptomatic and uncomplicated *P. falciparum* infection remains uncertain. Some studies indicate that blood group O reduces, but blood groups A or B increase the odds of uncomplicated *P. falciparum* infection [12, 13]. Another study reported lower odds of uncomplicated *P. falciparum infection* in individuals with blood group A or B as compared to those with blood group O [14]*.* Still others reported lack of relationship between ABO blood group and uncomplicated *P. falciparum* infection [15, 16]. Findings on the relationship of ABO blood group and asymptomatic *P. falciparum* infection also remains heterogeneous [17–19].

In addition, it is hypothesized that ABO blood group could affect susceptibility to placental *P. falciparum* infection, which is common among pregnant women in malaria endemic regions particularly among primiparous women with low immunity [20, 21]. However, study findings vary. Studies among pregnant women in Gambia and Malawi showed that prevalence of active placental *P. falciparum* infection increased in primiparous women with blood group O, but decreased in multiparous women with blood group O as compared to those with non-O blood groups [22, 23]. However, the odds of past placental *P. falciparum* infection was found to be greater among pregnant women with blood group O than those with non-O blood group in both primiparous and multiparous women in Sudan [24]. On the other hand, a study in Gabon showed similar odds of active placental *P. falciparum* infection in both primiparous and multiparous women with different blood groups [25].

Understanding the effect of ABO blood group on clinical manifestations of *P. falciparum* infection may contribute to the understanding of malaria pathogenesis and clinical morbidity. This in turn will facilitate investigation of antimalarial treatments and vaccines. Loscertales et al (2007), and Cserti and Dzik (2007) reviewed literature on the relationship between ABO blood group and *P. falciparum* infection published before 2007 [9, 10]. Many studies that report data on the relationship of ABO blood groups and *P. falciparum* infection have been published since 2007. However, the relationship of ABO blood group with asymptomatic, uncomplicated and placental *P. falciparum* infection remains unclear. Published data show that ABO blood group affects progression to severe malaria after infection with *P. falciparum*. While blood type A delays clearance of parasitized red blood cells (pRBCs) by promoting rosetting and cytoadherence, blood group O increases clearance of pRBC by reducing rosetting and cytoadherence [26–29]. Thus, we hypothesized that the prevalence or odds of asymptomatic *P. falciparum* infection would be greater, but the odds of uncomplicated *P. falciparum* infection lower in individuals with blood group A, B and AB compared to those with blood group O. The objective of this study was to systematically summarize literature on relationships between ABO blood group and asymptomatic, uncomplicated and placental *P. falciparum* infection published before February 4, 2017.

## Methods

The protocol for this review was registered in PROSPERO (ID = CRD42017068885), an international database of prospectively registered systematic reviews [30] and this report is accordance with the preferred reporting items for systematic reviews and meta-analyses (PRISMA) guidelines for systematic reviews (Additional file 1: Table S1. PRISMA Checklist) [31].

### Literature search strategies

Articles available in Pubmed, Embase, Web of Science, CINAHL and Cochrane Library were searched using the terms (“ABO blood type” OR “ABO blood group” OR “blood type” OR “blood group”) AND (*Plasmodium* OR malaria OR “*Plasmodium falciparum*” OR “*Plasmodium vivax*”) on February 04, 2017 (Additional file 2: Table S2. Literature search strategy). Language, date of inception, study design, age, gender and geography were not restricted. We examined references cited in reviews of malaria and ABO blood group for additional articles [9, 10]. Articles obtained from searches in the databases were combined in RefWorks. After the duplicates were removed, titles and abstracts of articles were screened, and full contents of eligible articles were reviewed. Two authors screened articles independently (AD and MG) using the eligibility criteria for the review. The degree of discrepancy in the screening and choice of articles between the two authors was very low and resolved by consensus.

### Eligibility criteria

All published original studies of any design except case studies that assessed association between ABO blood group and asymptomatic, uncomplicated, or placental *P. falciparum* infection were included. Studies that did not confirm *Plasmodium* infection by PCR, microscopy, rapid diagnostic test, or histology were excluded. In addition, studies were excluded if the type of *Plasmodium* species assessed was not falciparum or was not clearly stated. Moreover, studies were excluded from meta-analysis when they lacked sufficient data to estimate the association between ABO blood group and asymptomatic, uncomplicated, or placental *P. falciparum* infection.

### Outcome measures

Outcomes in this study were incidence or prevalence of asymptomatic, uncomplicated, or placental *P. falciparum* infection. Asymptomatic malaria refers to infections with *Plasmodium* parasite but without malaria-related symptoms such as fever, headache, nausea, chills, malaise, and sweating [5, 6]. Uncomplicated *P. falciparum* infection is accompanied by common malaria-related symptoms [3], but without severe malaria symptoms as defined by the World Health Organization [32]. Placental *P. falciparum* infection was defined as the presence of current (active) or past (passive) *Plasmodium* infection in the placenta as revealed by microscope or histology examination. Active placental *P. falciparum* infection refers to the presence of the parasite with or without parasite pigment [21]. Passive placental *P. falciparum* infection refers to the presence of parasite pigment without the parasite [21].

### Data collection

For each study, we extracted data on the study area, study year, sample size, study design, prevalence of *P. falciparum* infection, and method of malaria diagnosis among individuals with different blood groups. We also extracted data on the measures of association (odds ratio (OR) or relative risk (RR)) between the ABO blood group and asymptomatic, uncomplicated, or placental *P. falciparum* infection among individuals. Two authors extracted data independently (AD and MG). The degree of discrepancy in data extraction was minimal between the two authors. When there was discrepancy, it was resolved by consensus. When studies did not report adjusted OR or RR of *P. falciparum* infection, these values were calculated using raw data on the prevalence of *P. falciparum* infection among individuals with blood groups A, B, AB and O.

### Quality and bias assessment

We assessed quality of each study using six characteristics; selection bias, study design, confounder, blinding, data collection methods, withdrawal, and drop-outs following the scales suggested by the Effective Public Health Practice Project guidelines [33]. Each study was grouped as low, moderate, or high quality with respect to each of the six characteristics and an overall study quality was then determined based on the quality of all six characteristics. An overall quality of a study was grouped as high when the study has no weak rating with respect to each of the six characteristics, and moderate when the study has one weak rating in one of the six characteristics. An overall quality was graded as low when two or more weak ratings recorded out of the six characteristics.

### Data analysis

Meta-analyses were performed using Stata version 11 [34]. The summary ORs or RRs of *P. falciparum* infection and the corresponding 95% confidence interval (CI) values across different blood groups were estimated using Der Simonian and Laird method following a random effects model (Moran’s I^2^ ≥ 30%) or using the inverse variance method following a fixed effects model (Moran’s I^2^ < 30%) [35]. Publication bias was assessed using funnel plots and Egger’s asymmetry test (bias if *p* < 0.1) [36, 37]. The magnitude of heterogeneity across the studies was determined using Moran’s I^2^ and statistical significance was tested using Cochrane chi-square test based on the inverse-variance fixed-effect model [38]. Sub-group analysis of the studies, which assessed the relationship between ABO blood group and placental *P. falciparum* infection, was performed after grouping studies based on the type of placental *P. falciparum* infection (active versus (vs) passive) and parity (primiparous vs multiparous). Meta-regression was conducted to estimate association of study-specific ORs of *P. falciparum* infection by study regions (Africa, Asia, South America), age of the study participants (children, adult, all ages combined), and study design (cross-sectional, case control, cohort).

## Results

### Characteristics of the included studies

**Fig. 1:**
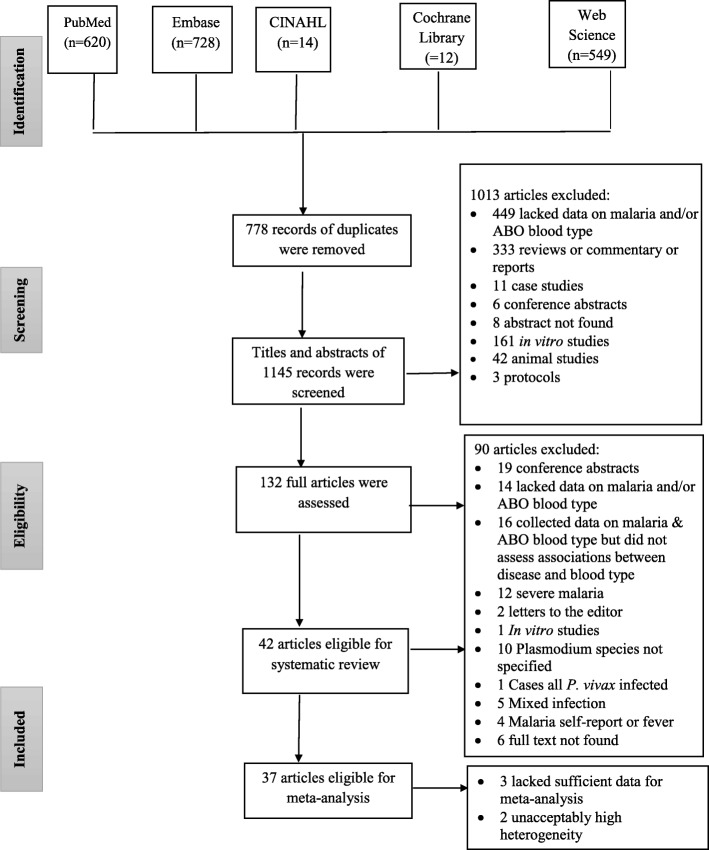
PRISMA flow diagram showing the number of articles retrieved, screened, excluded and included at each stage of the search

A total of 1923 articles were obtained after searching literature from five databases: Embase (*n* = 728), PubMed (*n* = 620), Web of science (*n* = 549), CINAHL (*n* = 14) and Cochrane Library (*n* = 12). Of the 1, 923 articles, 778 were found to be duplicates. After screening the titles and abstracts of the remaining 1145 articles, 132 were found eligible for full-text review. Ninety articles were excluded after full-text review based on inclusion/exclusion criteria. A total of 42 articles were included in this systematic review; 37 of them were also included in the meta-analysis (Fig. 1) [12–19, 22–25, 39–68]. Most (*n* = 30) studies were cross-sectional, seven studies were prospective cohort, and five were case-control in design. The 42 studies also differ in the age of the study population groups, 14 studies involved individuals of all age groups, 12 studies involved adolescents or adults, eight studies involved children and the remaining eight studies were conducted in pregnant women. Majority (*n* = 27) of the studies used microscope for the diagnosis of malaria. However, some studies, used both microscope and PCR (*n* = 6), histology (*n* = 4), rapid diagnostic (*n* = 1) or serology (n = 1) tests for the diagnosis of malaria. Still some studies used only PCR (n = 1) or histology technique (n = 2) for malaria diagnosis. (Additional file 3: Table S3. Characteristics of the studies).

### ABO blood group and uncomplicated *P. falciparum* infection

**Fig. 2:**
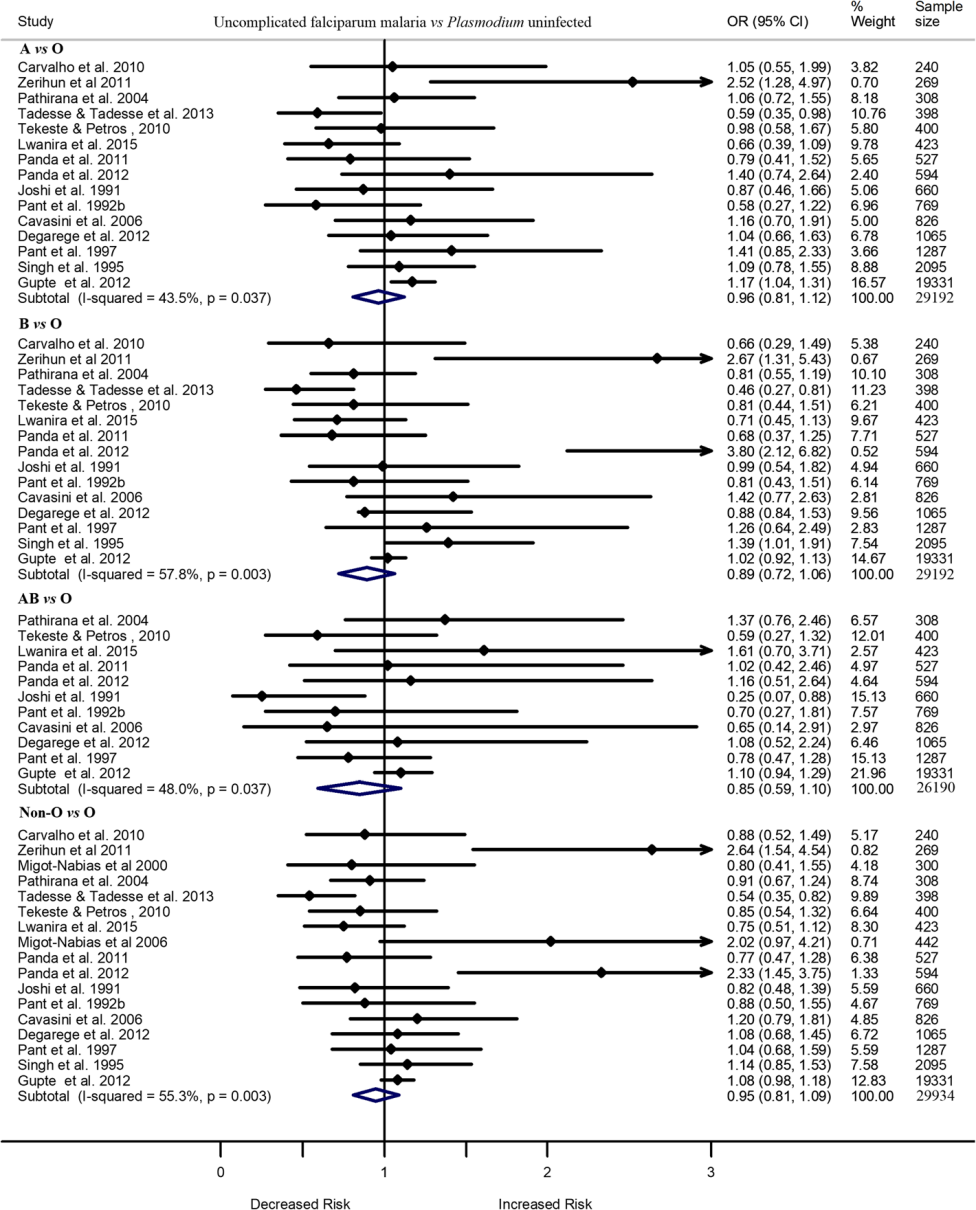
Forest plot showing the odds of uncomplicated *P. falciparum* infection in individuals with blood group A, B, AB or non-O vs those with blood group O

Of 42 included studies, 20 compared the odds of uncomplicated *P. falciparum* infection vs the odds of being uninfected with *Plasmodium*, and two studies compared the odds of uncomplicated *P. falciparum* infection vs the odds of developing asymptomatic *P. falciparum* infection among individuals with different blood group. Of the 20 studies, six reported significantly greater odds of uncomplicated *P. falciparum* infection, as compared to the odds of being not infected with the parasite, among individuals with blood group A [49, 66], B [12, 13, 64], AB [59], or non-O [12, 13] than those with blood group O. However, one study reported significantly lower odds of uncomplicated *P. falciparum* infection, as compared to being uninfected with *Plasmodium*, among individuals with blood group A, B, or non-O than those with blood group O [14]. The other 13 studies showed comparable odds of uncomplicated *P. falciparum* infection (vs uninfected with *Plasmodium*) among individuals with blood group A, B, AB, or non-O (A/B/AB) and those with blood group O. Meta-analysis of these studies showed that the odds of uncomplicated *P. falciparum* infection, as compared to the odds of being not infected with the parasite, did not differ significantly by blood group. All ORs were nonsignificant. They included: A vs O (OR 0.96, 95%CI 0.81–1.12, I^2^ 43.5%, 15 studies), B vs O (OR 0.89, 95%CI 0.72 –1.06, I^2^ 57.8%, 15 studies), AB vs O (OR 0.85, 95%CI 0.59 –1.10, I^2^ 48.0%, 10 studies), or non-O vs O (OR 0.95, 95%CI 0.81–1.09, I^2^ 55.3%, 17 studies) (Fig. 2).

Meta-analysis of two studies that compared the odds of uncomplicated *P. falciparum* infection vs asymptomatic *P. falciparum* infection also showed similar odds of uncomplicated *P. falciparum* infection among individuals with blood group A, B, or non-O and those of blood group O, but the odds of uncomplicated *P. falciparum* infection was lower in individuals with blood group AB than those of blood group O (OR 0.64, 95%CI 0.39 – 0.88, I^2^ 0.0%) [43, 56].

Out of the 20 studies, four were cohort studies [53–55, 64]. Meta-analysis of these four cohort studies showed similar risk of uncomplicated *P. falciparum* infection (vs uninfected with *Plasmodium*) among individuals with non-O blood groups and those with blood group O (RR 1.03, 95%CI 0.84 –1.22, I^2^ 57.3%) [53–55, 64]. Another study with a cohort design also reported similar risk of uncomplicated *P. falciparum* infection among children with blood group A, B, or AB as compared to those with blood group O, but this study was excluded from the meta-analysis due to lack of sufficient data [48]. Two other studies that reported data on the odds of uncomplicated *P. falciparum* infection among children by blood group were also not included in the meta-analysis because of wide 95% CI estimates (small number of cases), which introduced heterogeneity to the meta-analysis [59, 66].

### ABO blood group and asymptomatic *P. falciparum* infection

**Fig. 3:**
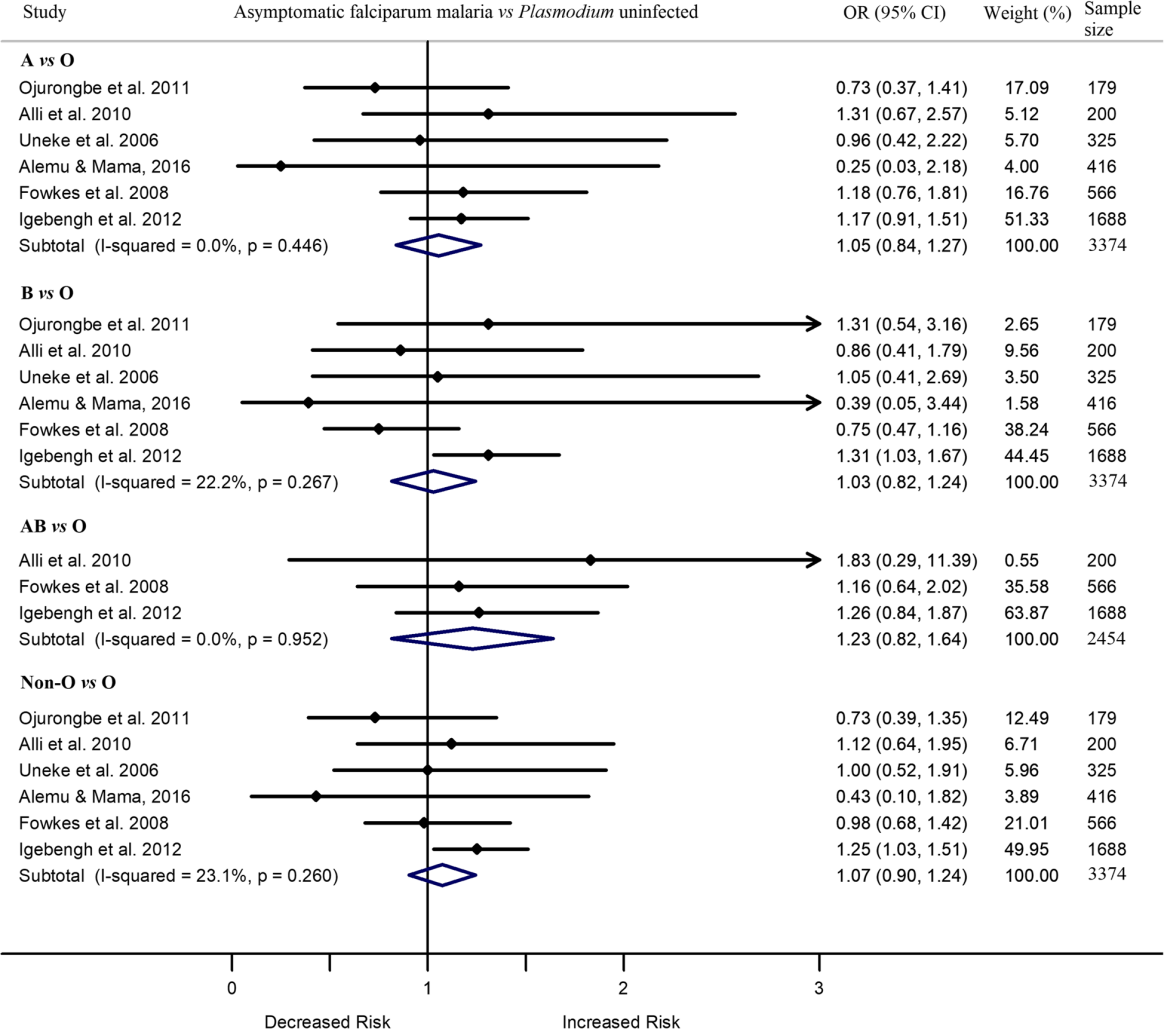
Forest plot showing the odds of asymptomatic *P. falciparum* infection in individuals with blood group A, B, AB or non-O vs those with blood group O

Out of 42 included studies, type of *P. falciparum* infection was assessed as asymptomatic in seven cross-sectional studies. Out of the seven, two studies in Nigeria reported contradictory results [18, 19]. Igebengh et al. 2012 [18] reported higher odds of asymptomatic *P. falciparum* infection in adults with blood group B or non-O than those with blood group O. However, Jeremiah et al. 2012 [19] reported lower odds of asymptomatic *P. falciparum* infection in children with blood group A, B, or non-O than those with blood group O. The remaining five studies showed similar odds of asymptomatic *P. falciparum* infection in children and adults with blood groups A, B, or AB and those with blood group O. Meta-analysis of the seven studies showed that individuals with asymptomatic infection had similar odds of having blood group A (OR 0.86, 95%CI 0.52 –1.20, I^2^ 63.4%), B (OR 0.84, 95%CI 0.44 –1.24, I^2^ 72.3%), AB (OR 1.21, 95%CI 0.82 –1.61, I^2^ 0.0%), or non-O (OR 0.85, 95%CI 0.51–1.19, I^2^ 78.5%) vs blood group O. The level of heterogeneity significantly decreased but the odds of asymptomatic *P. falciparum* infection remained similar between the comparison blood groups after removing one unique study [19], conducted in children. In these comparisons, ORs varied from 1.03 to 1.23 (A vs O [OR 1.05, 95%CI 0.84 –1.27, I^2^ 0.0%], B vs O [OR 1.03, 95%CI 0.82 –1.24, I^2^ 22.2%], AB vs O [OR 1.23, 95%CI 0.82 –1.64, I^2^ 0.0%] and all non-O vs O [OR 1.07, 95%CI 0.90 –1.24, I^2^ 23.1%]) (Fig. 3).

**Fig. 4:**
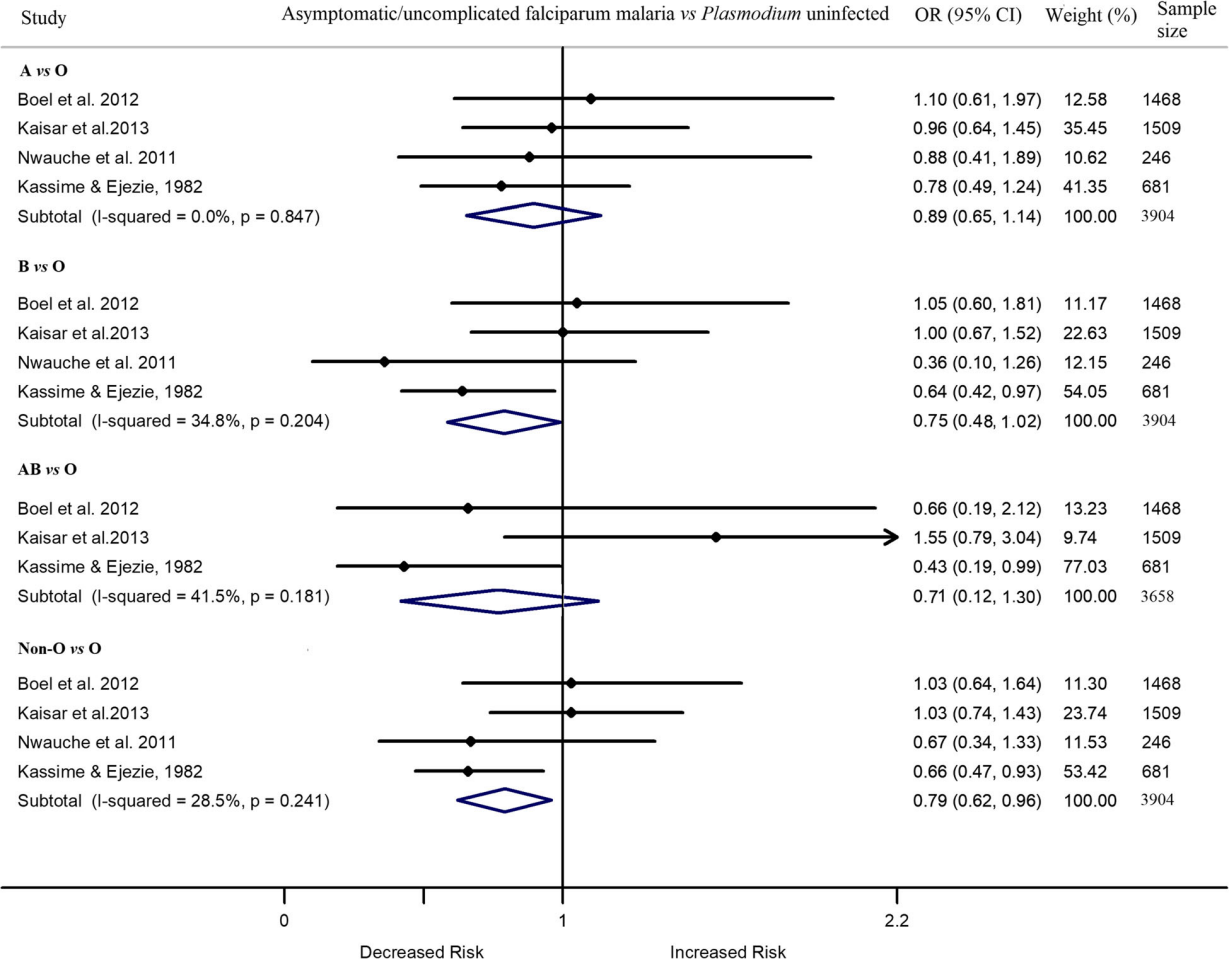
Forest plot showing the odds of asymptomatic/uncomplicated *P. falciparum* infection in individuals with blood group A, B, AB or non-O vs those with blood group O

Five studies assessed the relationship between asymptomatic and/or uncomplicated *P. falciparum* infection and ABO blood group without distinction between cases being asymptomatic or uncomplicated *falciparum* malaria [45, 51–53, 63]. Meta-analysis of the four studies showed a similar likelihood of asymptomatic/uncomplicated *P. falciparum* infection between individuals with blood group A, B, or AB and those with blood group O [45, 51–53] (Fig. 4). One study was excluded from the meta-analysis due to lack of sufficient data [63].

### ABO blood group and placental *P. falciparum* infection

Of the 42 studies, eight compared the prevalence of placental *P. falciparum* infection among women by blood group. When compared to blood group O, odds of active placental *falciparum* infection in blood group non-O was greater in primiparous women but lower in multiparous ones in Ghana [44]. In contrast, in a Malawi study, the odds of active placental *P. falciparum* infection was greater among non-O blood group than blood group O in multiaparous women, but lower among non-O blood group than blood group O in primiparous women [23]. In a Nigerian study, the odds of active placental *P. falciparum* infection was lower among pregnant women with non-O blood group than those with blood group O [67], but this difference was not significant when data were analyzed after stratifying based on parity. The odds of passive placental *P. falciparum* infection in a study in Gambia was greater among pregnant women with non-O blood group than those with blood group O [22]. However, four studies showed no significant difference in the odds of placental falciparum infection among pregnant women with blood groups A, B, or AB compared to those with non-O blood group [24, 25, 39, 42]. Meta-analysis based on the eight studies showed similar odds of placental *P. falciparum* infection as compared to those without placental *P. falciparum* infection among pregnant women with non-O blood group and those with blood group O (Table 1).

**Table 1:** Meta-analysis of the studies that compare the odds of placental *P. falciparum* infection among individuals with blood group A, B, AB or non-O vs those with blood type O

					Odds of Placental malaria (95% CI)		
Parity	Placental malaria Type	Study	Sample size	A vs O	B vs O	AB vs O	Non-O vs O
Primiparae or Multiparae	Active or passive vs none	Adam et al. 2007	293	0.8 (0.44, 1.44)	0.55 (0.27, 1.1)	0.57 (0.21, 1.55)	0.67 (0.41, 1.1)
		Loscertales & Brabin, 2006	198	1.35 (0.63, 2.91)	1.17 (0.59, 2.29)	1.58 (0.28, 9.01)	1.26 (0.71, 2.22)
		Senga et al. 2007	647	1.32 (0.89, 1.96)	1.22 (0.83, 1.79)	3.68 (1.39, 9.74)	1.35 (0.99, 1.85)
		Bedu-Addo et al. 2014	827	1.16 (0.81, 1.66)	1.62 (1.09, 2.39)	1.77 (0.8, 3.91)	1.38 (1.04, 1.85)
		Adegnika et al. 2011	378	1.11 (0.39, 3.19)	2.38 (0.91, 6.23)	2.00 (0.23, 17.13)	1.64 (0.75, 3.59)
		Ukaga et al. 2007	586	0.72 (0.46, 1.13)	0.68 (0.39, 1.18)	0.21 (0.07, 0.62)	0.61 (0.42, 0.89)
		Adam et al. 2009	236	–	–	–	1.25 (0.91, 2.5)
		Alim et al. 2015	126	–	–	–	1.11 (0.4, 3.33)
		summary OR (95% CI)		0.97 (0.75, 1.19) I^2^ = 8.2%, *p* = 0.007	1.02 (0.64, 1.40) I^2^ = 58.0%, *p* = 0.036	0.59 (0.02, 1.14) I^2^ = 32.9%, *p* = 0.189	1.01 (0.73, 1.29) I^2^ = 58.8%, *p* = 0.017
Primiparae	Active vs uninfected	Adam et al. 2007	293	2/31 vs 0/51	0/24 vs 0/51	1/8 vs 0/51	3/50 vs 0/51
		Loscertales & Brabin, 2006	198	0.55 (0.15, 1.99)	0.33 (0.10, 1.08)	–	0.41 (0.16, 1.08)
		Senga et al. 2007	647	0.31 (0.10, 0.97)	0.79 (0.30, 2.06)	0.93 (0.06, 15.62)	0.54 (0.24, 1.21)
		Bedu-Addo et al. 2014	827	2.59 (1.03, 6.50)	1.56 (0.84, 2.91)	4.53 (0.52, 39.74)	1.90 (1.10, 3.28)
		Adegnika et al. 2011	378	4.8 (0.73, 31.48)	1.85 (0.16, 2.2)	-	3.2 (0.55, 18.56)
		summary OR (95% CI)		0.41 (0.003, 0.82) I^2^ = 1.4%, *p* = 0.385	1.04 (0.3, 1.78) I^2^ = 68.9%, *p* = 0.022	1.42 (−5.81, 8.65) I^2^ = 0.0%, *p* = 0.738	0.75 (0.17, 1.34) I^2^ = 53.8%, *p* = 0.090
	Passive vs uninfected	Adam et al. 2007	293	0.46 (0.15, 1.41)	0.31 (0.08, 1.20)	0.87 (0.15, 5.00)	0.44 (0.18, 1.08)
		Loscertales & Brabin, 2006	198	2.13 (0.42, 10.73)	1.28 (0.28, 5.93)	–	2.00 (0.55, 7.18)
		Senga et al. 2007	647	1.28 (0.58, 2.81)	1.24 (0.54, 2.83)	2.48 (0.26, 23.38)	1.32 (0.67, 2.52)
		Bedu-Addo et al. 2014	827	–	–	–	2.23 (1.17, 4.26)
		summary OR (95% CI)		0.68 (0.13, 1.22) I^2^ = 0.0%, *p* = 0.390	0.59 (0.03 1.21) I^2^ = 14.4%, *p* = 0.331	0.94 (0.44, 3.31) I^2^ = 0.0%, *p* = 0.789	0.82 (0.08, 1.57) I^2^ = 42.5%, *p* = 0.176
	Active vs passive	Loscertales & Brabin, 2006	198	0.26 (0.05, 1.26)	0.63 (0.27, 1.49)	0.38 (0.04, 3.53)	0.42 (0.2, 0.86)
		Senga et al. 2007	647	0.24 (0.08, 0.70)	0.69 (0.31, 1.53)	0.49 (0.05, 4.34)	0.46 (0.23, 0.92)
		Bedu-Addo et al. 2014	827	–	–	–	1.44 (0.56, 3.69)
		summary OR (95% CI)		0.24 (0.03, 0.52), I^2^ = 0.0%, *p* = 0.954	0.66 (0.23, 1.09), I^2^ = 0.0%, *p* = 0.892	0.42 (0.01, 1.78), I^2^ = 0.0%, *p* = 0.938	0.46 (0.23, 0.69) I^2^ = 0.0%, *p* = 0.458
Multiparae	Active vs uninfected	Adam et al. 2007	293	3.59 (0.66, 19. 69)	2 (0.26, 15.12)	–	2.37 (0.47, 11.91)
		Loscertales & Brabin, 2006	198	1.55 (0.45, 5.34)	2.07 (0.72, 5.93)	2.07 (0.26, 16.27))	1.87 (0.77, 4.56)
		Senga et al. 2007	647	1.78 (0.95, 3.35)	1.54 (0.84, 2.86)	5.27 (1.44, 19.32)	1.78 (1.07, 2.96)
		Bedu-Addo et al. 2014	827	0.21 (0.11, 0.40)	0.36 (0.19, 0.66)	0.36 (0.12, 1.07)	0.29 (0.17, 0.48)
		Adegnika et al. 2011	378	0.51 (0.11, 2.37)	2.59 (0.91, 7.37)	2.44 (0.27, 21.98)	1.36 (0.55, 3.34)
		summary OR (95% CI)		0.76 (0.02, 1.54) I^2^ = 51.7%, *p* = 0.082	1.11 (0.15, 2.07) I^2^ = 52.2%, *p* = 0.079	0.38 (0.09, 0.86) I^2^ = 0.0%, *p* = 0.515	1.20 (0.22, 2.19) I^2^ = 72.1%, *p* = 0.006
	Passive vs uninfected	Adam et al. 2007	293	0.56 (0.19, 1.66)	0.99 (0.37, 2.62)	0.47 (0.10, 1.94)	0.70 (0.32, 1.53)
		Loscertales & Brabin, 2006	198	3.22 (0.94, 11.05)	2.93 (0.94, 9.13)	–	2.76 (1.04, 7.34)
		Senga et al. 2007	647	1.22 (0.58, 2.58)	1.18 (0.58, 2.39)	6.32 (1.71, 23.41)	1.38 (0.78, 2.45)
		summary OR (95% CI)		0.82 (0.24, 1.41) I^2^ = 0.0%, *p* = 0.375	1.16 (0.46, 1.85) I^2^ = 0.0%, *p* = 0.668	1.01 (0.001, 2.77) I^2^ = 16.1%, *p* = 0.304	1.06 (0.38, 1.74) I^2^ = 31.8%, *p* = 0.231
	Active vs passive	Loscertales & Brabin, 2006	198	1.46 (0.6, 3.54)	0.71 (0.21, 2.34)	**–**	0.68 (0.24, 1.95)
		Senga et al. 2007	647	1.46 (0.6, 3.54)	1.31 (0.56, 3.08)	**–**	1.29 (0.64, 2.59)
		Adam et al. 2007	293	5.18 (0.76, 21.87)	2.9 (0.36, 26.39)	**–**	1.21 (0.16, 9.23)
		summary OR (95% CI)		1.49 (0.46, 2.53) I^2^ = 0.0%, *p* = 0.790	0.97 (0.16, 1.78) I^2^ = 0.0%, *p* = 0.743	–	0.95 (0.31, 1.59) I^2^ = 0.0%, *p* = 0.650

Summary OR estimated using a meta-analysis technique applying fixed (I^2^ < 30%) or random (I^2^ ≥ 30%) effect model. Statistical significance of the heterogeneity of the studies was tested using the Cochran’s Q test at α = 5%

Subgroup analysis based on parity and nature of placental *P. falciparum* infection showed lower odds of active placental *P. falciparum* infection (compared to absence of placental *P. falciparum* infection) among women with blood group A than those with blood group O in primiparous women (OR 0.41, 95%CI 0.003 – 0.82, I^2^ 1.4%, four studies). The difference in odds of active placental *P. falciparum* infection between blood group A (OR 0.24, 95%CI 0.03 – 0.52, I^2^ 0.0%; 2 studies) or non-O blood group (OR 0.46, 95%CI 0.23 – 0.69, I^2^ 0.0%, three studies) and blood group O in primiparous women remained significant even when the comparison groups were women with passive placental *P. falciparum* infection. However, the difference in the odds of passive placental *P. falciparum* infection (as compared to without placental *P. falciparum* infection) among women with blood group O and those with blood group A, B, or AB were not significant in both primiparous and multiparous women (Table 1).

### Publication bias and source of heterogeneity

The funnel plots based on the odds ratio of uncomplicated *P. falciparum* infection (vs uninfected) and the corresponding standard errors among individuals with blood group A vs O, B vs O, AB vs O and non-O vs O were symmetrical (Additional file 4: Figure S1. Funnel plots). The Egger’s test for the asymmetry was not significant for all the comparisons blood groups; A vs O (*p* = 0.234), B vs O (*p* = 0.932), AB vs O (*p* = 0.162) and non-O vs O (*p* = 0.846). Similarly, the funnel plots that showed the odds ratio of asymptomatic *P. falciparum* infection and the corresponding standard errors among individuals with blood group A vs O, B vs O and AB vs O were symmetrical. The corresponding Egger’s tests were non-significant for all the comparison blood groups except for blood group non-O vs O (Additional file 5: Figure S2. Funnel plots). The Egger’s test for asymmetry of funnel plots that showed the OR of placental *P. falciparum* infection among pregnant women with blood group A vs O (*p* = 0.911), B vs O (*p* = 0.666), AB vs O (*p* = 0.917) and non-O vs O (*p* = 0.991) were all nonsignificant (Additional file 6: Figure S3. Funnel plots).

The OR of uncomplicated *P. falciparum* among individuals with blood group A, B, AB or non-O vs those with blood group O did not vary by age of study participants, study region, or design of the study. Similarly, OR of asymptomatic infection among individuals with blood group A, B, AB or non-O vs those with blood group O did not vary with age of study participants, study region or and study design (Additional file 7: Table S4. Meta regression test values).

### Quality of the studies

Most studies reported high quality data-collection methods and control for confounders. Many studies followed procedures of moderate quality for recruitment of study participants. However, most studies had low quality study design (cross-sectional). Overall quality of the studies using six characteristics---- selection bias, study design, confounder, blinding, data collection methods, withdrawal and dropouts showed that nine studies were of strong quality, six studies were moderate quality and 27 studies were low quality (Table 2).

**Table 2:** Assessment of the quality of all studies included in the review

Study no.	Author, Year [References]	Selection bias	Study design	Confounders	Blinding	Data collection methods	Withdrawals and drop-outs	Final rating
1	Adam et al. 2009 [39]	2	3	3	2	1	NA	3
2	Adam et al. 2007 [24]	2	3	3	2	1	NA	3
3	Adegnika et al. 2011 [25]	2	3	3	2	1	NA	3
4	Alemu and Mama, 2016 [40]	2	3	3	2	1	NA	3
5	AlIi et al. 2010 [41]	2	3	3	2	1	NA	3
6	Alim et al. 2015 [42]	2	3	3	2	1	NA	3
7	Amodu et al. 2012 [43]	2	3	3	2	1	NA	3
8	Bedu-Addo et al. 2014 [44]	2	3	3	2	1	NA	3
9	Boel et al. 2012 [45]	1	1	2	2	1	2	1
10	Carvalho et al. 2010 [15]	1	2	2	2	1	2	1
11	Cavasini et al. 2006 [46]	1	2	2	2	1	2	1
12	Degarege et al. 2012 [16]	2	3	1	1	2	2	1
13	Fowkes et al. 2008 [47]	2	3	3	2	2	NA	3
14	Giha et al. 2000 [48]	2	1	3	2	1	2	2
15	Gupte et al. 2012 [49]	2	2	3	2	2	2	2
16	Igbeneghu et al. 2012 [18]	2	3	3	2	1	NA	3
17	Jeremiah et al. 2012 [19]	2	3	3	2	1	NA	3
18	Joshi et al. 1987 [50]	2	3	3	2	1	NA	3
19	Kaisar et al. 2013 [51]	1	3	2	2	1	NA	2
20	Kassime & Ejezie, 1982 [52]	2	3	3	2	1	NA	3
21	Loscertales & Brabin, 2006 [22]	2	3	3	2	1	NA	3
22	Lwanira et al. 2015 [53]	1	1	1	2	2	1	1
23	Migot-Nabias et al. 2000 [54]	2	1	1	2	1	2	1
24	Migot-Nabias et al. 2006 [55]	2	1	1	2	1	2	1
25	Missinou et al. 2003 [56]	2	1	2	2	1	2	1
26	Nwauche et al. 2011 [57]	2	3	3	2	1	NA	3
27	Ojurongbe et al. 2011 [17]	2	3	3	2	1	NA	3
28	Panda et al. 2012 [12]	2	3	2	2	1	NA	2
29	Panda et al. 2011 [58]	2	3	2	2	1	NA	2
30	Pant et al. 1992a [59]	2	3	3	2	1	NA	3
31	Pant et al. 1992b [60]	2	3	3	2	1	NA	3
32	Pant et al. 1997 [61]	2	3	3	2	1	NA	3
33	Pathirana et al. 2004 [62]	2	3	3	1	1	NA	3
34	Rabha et al. 2012 [63]	2	3	3	2	1	NA	3
35	Senga et al. 2007 [23]	2	3	3	2	2	NA	3
36	Singh et al. 1995 [64]	2	1	2	2	1	2	1
37	Tadesse & Tadesse, 2013 [14]	2	3	3	2	1	NA	3
38	Tekeste & Petros, 2010 [65]	2	3	3	2	1	NA	3
39	Thakur & Verma, 1992 [66]	2	3	3	2	1	NA	3
40	Ukaga et al. 2007 [67]	2	3	3	2	1	NA	3
41	Uneke et al. 2006 [68]	2	3	3	2	1	NA	3
42	Zerihun et al. 2011 [13]	2	3	3	2	1	NA	3

1 = strong; 2 = moderate; 3 = weak; NA = not applicable

## Discussion

The current meta-analysis showed lack of association of ABO blood group with prevalence or incidence of asymptomatic and/or uncomplicated *P. falciparum* infection. This suggests that the ABO blood group may not affect susceptibility to asymptomatic and/or uncomplicated *P. falciparum* infection. A meta-analysis by Taylor et al. (2012) also confirmed lack of effect of different human red blood cell polymorphism (e.g. hemoglobin S, hemoglobin C, α and β thalassemia) on susceptibility to asymptomatic and uncomplicated malaria [69]. Indeed, there is no substantial research evidence that confirms any influence of ABO blood group on human contact with mosquitoes. Two older (and one more recent) studies reported potential biting preference of *Anopheles gambae* [70, 71] and *Aedes albopictus* [72] mosquitos for blood group O [70–72]. However, most findings suggest that ABO blood group affects pathogenesis of malaria after the parasite enters the human body [9–11, 28]. Thus, effect of the ABO blood group on uncomplicated *P. falciparum* infection would be better investigated with comparison groups consisting of asymptomatic falciparum malaria cases rather than who are uninfected (or healthy normal) with *Plasmodium*. Only two studies included in this review compared odds of uncomplicated *P. falciparum* infection to asymptomatic *P. falciparum* infection by blood groups [43, 56].

On the other hand, the odds of active placental *P. falciparum* infection was lower in primiparous women with non-O blood group particularly in those with blood group A, than in those with blood group O. This suggests that primiparous women with blood group O may be at increased risk and that of blood group A could be protective against active placental *P. falciparum* infection. A meta-analysis of three studies by Adegnika et al. (2011) also showed increased odds of active placental *P. falciparum* infection in primiparous women with blood group O than non-O blood group [25]. Maternal blood group may affect placental *P. falciparum* infection in a counterintuitive way. It may be that mothers with blood group A (if they are A1) are more likely to sequester their infected red cells in the maternal circulation (due to more cytoadhesion) leaving less infected red cells to passively reach the placenta [26]. Whether those *P. falciparum* erythrocytes membrane protein (PfEMP-1) positive red cells that do reach the placenta remain in the placenta or not would seem to be affected by placental gene expression. Indeed, studies showed association between peripheral parasitaemia and placental malaria infection during pregnancy [73, 74]. Maternal blood group may also affect maternofetal antibody transfer efficiency. Transfer efficiency of IgG1 and IgG3 against placental *P. falciparum* infection from mother to the fetus might be reduced in primiparous women with blood group O than those with blood group A. A recent study confirmed association of placental *P*. *falciparum* infection with reduced transfer efficiency of IgG1 and IgG3 from primiparous mother to the fetus, but this association was not seen in multiparous women [75]. Moreover, the increased occurrence of active placental *P. falciparum* infection among primiparous women with blood group O might be related to other genetic factors linked to ABO gene at chromosome 9 (9q34.2; especially to O alleles) that could act as permissive factors to active placental *P. falciparum* infection. Ordi et al (1998) reported increased placental lesions, characterized by accumulation of inflammatory infiltrate in the intervillous space, among primiparous women with placental malaria [76].

This study has implication for research. The reasons for the finding of increased odds of active placental *P. falciparum* infection among individuals with blood group O than those with blood group non-O is unclear. Additional studies are essential to understanding of mechanisms by which maternal O blood group increases the risk of active placental *P. falciparum* infection, while blood group A protects against it. Future studies may characterize and evaluate the nature of antigens on erythrocyte membranes of different blood types which would increase or decrease the risk of active placental *P. falciparum* infection.

This is the first systematic review and meta-analysis to assess the relationship of ABO blood group with asymptomatic and uncomplicated *P. falciparum* infection. In addition, although a previous study estimated the relationship between ABO blood group and placental *P. falciparum* infection [23] using four studies, the current study summarizes that relationship using eight studies. Moreover, there was no publication bias among the studies which compared the odds of asymptomatic, uncomplicated *P. falciparum* infection among individuals with blood group A, B, AB or Non-O and those with blood group O. However, this review has some limitations. There was a moderate level of heterogeneity in some of the meta-analyses performed. Variations in the age of the study participants, and study regions and designs did not significantly contribute to the increased heterogeneity. In addition, studies included in this review compared the odds of uncomplicated and/or asymptomatic *P. falciparum* infection with the odds of being uninfected with *Plasmodium*. Thus, it is impossible to confirm fully if the ABO blood group can affect progression from asymptomatic to uncomplicated *P. falciparum* infection. Future studies may compare the odds of uncomplicated *P. falciparum* infection with the odds of having asymptomatic *P. falciparum* infection among individuals with different blood groups. Moreover, limitations in the original studies could have affected the current summary estimates. For example, some studies that investigated relationship of ABO blood group with placental *P. falciparum* infection involved small sample size or few cases, thus had very wide confidence interval for the effect measure estimates and low statistical power to reject false associations. In addition, most studies included in the review did not control for confounders (e.g. socioeconomic factors, nutrition, infection, thalassemias, and haemoglobin variants including HbS, HbC and HbE) while they evaluated the relationship of ABO blood group with *P. falciparum* infection [77–82].

## Conclusions

This review suggests that primiparous women with blood group O appear to be more susceptible to active placental *P. falciparum* infection than those with non-O blood group. However, ABO blood group may not influence susceptibility to asymptomatic and uncomplicated *P. falciparum* infection. Future studies need to investigate the mechanisms by which blood group A reduces risk of active placental falciparum infection in primiparous women.

## Additional files

Additional file 1: **Table S1.** PRISMA checklist. (DOC 65 kb)

Additional file 2: **Table S2.** Literature search strategy (DOCX 13 kb)

Additional file 3: **Table S3.** Characteristics of the studies included in this review (DOCX 42 kb)

Additional file 4: **Figure S1.** Funnel plot. Odds ratio against standard error of odds ratio for studies, which compared the odds of uncomplicated *Plasmodium falciparum* infection vs *Plasmodium* uninfected among individuals with blood group A vs O, B vs O, AB vs O and Non-O vs O. (DOCX 279 kb)

Additional file 5: **Figure S2.** Funnel plot. Odds ratio against standard error of odds ratio for studies, which compared the odds of asymptomatic *Plasmodium falciparum* infection vs *Plasmodium* uninfected among individuals with blood group A vs O, B vs O, AB vs O and Non-O vs O. (DOCX 244 kb)

Additional file 6: **Figure S3.** Funnel plot. Odds ratio against standard error of odds ratio for studies, which compared the odds of placental *Plasmodium falciparum* infection vs *Plasmodium* uninfected among individuals with blood group A vs O, B vs O, AB vs O and Non-O vs O. (DOCX 205 kb)

Additional file 7: **Table S4.** Sources of heterogeneity assessment based on meta-regression analyses. (DOCX 16 kb)

## Abbreviations

CI: Confidence interval
OR: Odds ratio
PRISMA: Preferred reporting items for systematic reviews and meta-analyses
PROSPERO: International prospective register of systematic reviews
RR: Relative risk
