# Supplementary material for: Effect of ABO blood group on asymptomatic, uncomplicated and placental Plasmodium falciparum infection: systematic review and meta-analysis
Source: BMC Infect Dis. 2019 Jan 25;19:86. doi: 10.1186/s12879-019-3730-z (PMC6346527; doi:10.1186/s12879-019-3730-z)
Supplement: Supplementary file 2 — Table S2. Literature search strategy (DOCX 13 kb) [file 12879_2019_3730_MOESM2_ESM.docx]

Additional file 2: Table S2. Literature search strategy

| **PubMed** |
| --- |
| ("ABO blood type"[All Fields] OR "ABO blood group"[All Fields] OR "blood type"[All Fields] OR "blood group"[All Fields]) AND (("plasmodium"[MeSH Terms] OR "plasmodium"[All Fields]) OR ("malaria"[MeSH Terms] OR "malaria"[All Fields]) OR "Plasmodium falciparum"[All Fields] OR "Plasmodium vivax"[All Fields]) |
| **Embase** |
| 'abo blood type' OR 'abo blood group'/exp OR 'abo blood group' AND ('plasmodium'/exp OR plasmodium OR 'malaria'/exp OR malaria OR 'plasmodium falciparum'/exp OR 'plasmodium falciparum') |
