# Supplementary material for: Effect of ABO blood group on asymptomatic, uncomplicated and placental Plasmodium falciparum infection: systematic review and meta-analysis
Source: BMC Infect Dis. 2019 Jan 25;19:86. doi: 10.1186/s12879-019-3730-z (PMC6346527; doi:10.1186/s12879-019-3730-z)
Supplement: Supplementary file 3 — Table S3. Characteristics of the studies included in this review (DOCX 42 kb) [file 12879_2019_3730_MOESM3_ESM.docx]

Additional file 3: Table S3. Characteristics of the studies included in this review

| Reference (Study area) | Age group | Sample size | Study design | Prevalence *P. falciparum* | Type of malaria | Findings | Diagnostic techniques |
| --- | --- | --- | --- | --- | --- | --- | --- |
| Alemu & Mama, 2016 (Ethiopia) | 22 ± 0.29 | 416 | CS | 4.1% | Asymptomatic | A *vs* O (OR 0.25, 95% CI 0.03 – 2.18) B *vs* O (OR 0.39, 95% CI 0.05 – 3.44) AB *vs* O (OR 2.0, 95% CI 0.22 – 18.13) Non-O *vs* O (OR 0.43, 95% CI 0.10 – 1.82) | Microscopy |
| Alli et al. 2010 (Nigeria) | 18-65 | 200 | CS | 46.5% | Asymptomatic | A *vs* O (OR 1.31, 95% CI 0.67 – 2.57) B *vs* O (OR 0.86, 95% CI 0.41 – 1.79) AB *vs* O (OR 1.83, 95% CI 0.29 – 11.39) Non-O *vs* O (OR 1.12, 95% CI 0.64 – 1.95) | Microscopy |
| Amodu et al. 2012 (Nigeria) | Children | 3100 | CS | All malaria positive | Uncomplicated *vs* Asymptomatic | A *vs* O (OR 0.94, 95% CI 0.76 – 1.16) B *vs* O (OR 1.08, 95% CI 0.88 – 1.33) AB *vs* O (OR 0.63, 95% CI 0.43 – 0.93) Non-O *vs* O (OR 0.97, 95% CI 0.82 – 1.14) | Microscopy |
| Boel et al. 2012 (Thailand) | Pregnant women | 1468 | PC | 13.0% | Asymptomatic/ Uncomplicated | A *vs* O (OR 1.10, 95% CI 0.61– 1.97) B *vs* O (OR 1.05, 95% CI 0.60 – 1.81) AB *vs* O (OR 0.66, 95% CI 0.19 – 2.12) Non-O *vs* O (OR 1.03, 95% CI 0.64 – 1.64) A *vs* O (RR 1.09, 95% CI 0.64 – 1.88) B *vs* O (RR 1.04, 95% CI 0.63 – 1.73) AB *vs* O (RR 0.67, 95% CI 0.21 – 2.14) Non-O *vs* O (RR 1.03, 95% CI 0.67 – 1.59) Lack of significant difference in *Plasmodium* density between individuals with different blood group | Microscopy |
| Cavasini et al. 2006 (Brazil) | 28±8 | 826 | CC | 14.6% | Uncomplicated | A *vs* O (OR 1.16, 95% CI 0.7 – 1.91) B *vs* O (OR 1.42, 95% CI 0.77 – 2.63) AB *vs* O (OR 0.65, 95% CI 0.14 – 2.91) Non-O *vs* O (OR 1.20, 95% CI 0.79 – 1.81) | Microscopy |
| Degarege et al. 2012 (Ethiopia) | All ages | 1065 | CS | 13% | Uncomplicated | A *vs* O (OR 1.04, 95% CI 0.66 – 1.63) B *vs* O (OR 0.88, 95% CI 0.84 – 1.53) AB *vs* O (OR 1.08, 95% CI 0.52 – 2.24) Non-O *vs* O (OR 1.08, 95% CI 0.68 – 1.45) Anaemia A *vs* O (OR 2.82, 95% CI 1.05 – 7.56) Hemoglobin A *vs* O (β −1.25, 95% CI −2.31 – −0.19) Anaemia O *vs* non-O (aOR 0.58, 95% CI 0.26 – 1.31) No difference in *Plasmodium* density between blood groups | Microscopy |
| Fowkes et al. 2008 (Papua New Guinea) | 1-17 | 555 | CS | 58.7% | Asymptomatic | A *vs* O (OR 1.18, 95% CI 0.76 – 1.81) B *vs* O (OR 0.75, 95% CI 0.47 – 1.16) AB *vs* O (OR 1.08, 95% CI 0.58 – 2.02) Non-O *vs* O (OR 0.98, 95% CI 0.68 – 1.42) | Microscopy |
| Giha et al. 2000 (Sudan) | All ages | 457 | PC |  | Uncomplicated | The risk of getting infection was similar between individuals with blood type O *vs* A, O *vs* B, O *vs* AB | Microscope |
| Gupte et al. 2012 (India) | Adolescents & adults | 19331 | CC | 22.4% | Uncomplicated | A *vs* O (OR 1.17, 95% CI 1.04 – 1.31) B *vs* O (OR 1.02, 95% CI 0.92 – 1.13) AB *vs* O (OR 1.10, 95% CI 0.94 – 1.29) Non-A *vs* O (OR 1.08, 95% CI 0.98 – 1.18) | Microscopy |
| Igebengh et al. 2012 (Nigeria) | Adults (≥16 years) | 1688 | CS | 44.1% | Asymptomatic | A *vs* O (OR 1.17, 95% CI 0.91 – 1.51) B *vs* O (OR 1.31, 95% CI 1.03 – 1.67) AB *vs* O (OR 1.26, 95% CI 0.84 – 1.87) Non-O *vs* O (OR 1.25, 95% CI 1.03 – 1.51) Parasite density was higher in A than B and O | Microscopy |
| Jeremiah et al. 2012 (Nigeria) | 1-9 | 240 | CS | 27.5% | Asymptomatic | A *vs* O (OR 0.37, 95% CI 0.17 – 0.83) B *vs* O (OR 0.32, 95% CI 0.15 – 0.72) AB *vs* O (OR 0.87, 95% CI 0.21 – 3.63) Non-O *vs* O (OR 0.38, 95% CI 0.21 – 0.69) | Microscopy |
| Joshi et al. 1987(India) | 10-60 | 660 | CS | 13.8% | Uncomplicated | A *vs* O (OR 0.87, 95% CI 0.46 – 1.66) B *vs* O (OR 0.99, 95% CI 0.54 – 1.82) AB *vs* O (OR 0.25, 95% CI 0.07 – 0.88) Non-O *vs* O (OR 0.82, 95% CI 0.48 – 1.39) | Microscopy |
| Kassime & Ejezie, 1982 | 7-14 | 681 | CS | 39.5% | Asymptomatic/ uncomplicated | A *vs* O (OR 0.78, 95% CI 0.49 – 1.24) B *vs* O (OR 0.64, 95% CI 0.42 – 0.97) AB *vs* O (OR 0.43, 95% CI 0.19 – 0.99) Non-O *vs* O (OR 0.66, 95% CI 0.47 – 0.93) | Microscopy |
| Lwanira et al. 2015 (Uganda) | ½-9 | 423 | PC | 49% | Uncomplicated | A *vs* O (OR 0.66, 95% CI 0.39 – 1.09) B *vs* O (OR 0.71, 95% CI 0.45 – 1.13) AB *vs* O (OR 1.61, 95% CI 0.70 – 3.71) Non-O *vs* O (OR 0.75, 95% CI 0.51– 1.12)  A *vs* O (RR 0.69, 95% CI 0·52 – 0.93) B *vs* O (RR 0.74, 95% CI 0.58 – 0.95) AB *vs* O (RR 1.17, 95% CI 0.87 – 1.57) Non-O *vs* A (RR 0.77, 95% CI 0.64 – 0.94) | Microscopy |
| Migot-Nabias et al. 2000 (Gabon) | 9.50 ±2.65 | 300 | PC |  | Uncomplicated | Non-O *vs* O (OR 0.80, 95% CI 0.41 – 1.55) Non-O *vs* O (RR 0.89, 95% CI 0.64 –1.25) Lower parasite density in O (mean density= 0.87, 95% CI 0.74–1.03) than non-O (mean density= 1.15; 95% CI 0.93–1.43) (p=0.043) | Microscopy |
| Migot-Nabias et al. 2006 (Sengal) | 4.9 (±1.6) | 442 | PC |  | Uncomplicated  Asymptomatic | Non-O *vs* O (aOR 2.02, 95% CI 0.97 – 4.21) Non-O *vs* O (RR 1.25, 95% CI 1.01 – 1.53) Higher asymptomatic parasite density in non-O than O (regression coefficient: 0.03, p=0.07) | Microscopy |
| Missinou et al. 2003 (Gabon) | 3 & 1/2±2.4 | 200 | PC | Mean infection rate, 1.5 infection/child/year | Uncomplicated *vs* Asymptomatic | A *vs* O (OR 1.03, 95% CI 0·43 – 2.45) B *vs* O (OR 0.73, 95% CI 0.33 – 1.63) AB *vs* O (OR 0.77, 95% CI 0.22 – 2.69) Non-O *vs* O (OR 0.85 95% CI 0.45 – 1.58)  A *vs* O (RR 1.00, 95% CI 1.95 – 1.06) B *vs* O (RR 0.98, 95% CI 0.92 – 1.04) AB *vs* O (RR 0.98, 95% CI 0.89 – 1.08) Non-O *vs* O (RR 0.99, 95% CI 0.95 –1.03) | Microscopy |
| Pant et al. 1992a (India) | All ages | 783 | CS | 4.0% | Uncomplicated | A *vs* O (OR 2.44, 95% CI 0.82 – 7.29) B *vs* O (OR 1.15, 95% CI 0.38 – 3.48) AB *vs* O (OR 4.2, 95% CI 1.29 – 13.68) Non-O *vs* O (OR 1.91, 95% CI 0.72 – 5.02) | Microscopy |
| Pant et al. 1992b (India) | All ages | 769 | CS | 7.9% | Uncomplicated | A *vs* O (OR 0.58, 95% CI 0.27 – 1.22) B *vs* O (OR 0.81, 95% CI 0.43 – 1.51) AB *vs* O (OR 0.7, 95% CI 0.27 – 1.81) Non-O *vs* O (OR 0.88, 95% CI 0.50 – 1.55) | Microscopy |
| Pant et al. 1997 (India) | All ages | 1287 | CS | 8.9% | Uncomplicated | A *vs* O (OR 1.41, 95% CI 0.85 – 2.33) B *vs* O (OR 1.26, 95% CI 0.64 – 2.49) AB *vs* O (OR 0.78, 95% CI 0.47 – 1.28) Non-O *vs* O (OR 1.04, 95% CI 0.68 – 1.59) | Microscopy |
| Pathirana et al. 2004 (Sirlanka) | 1 to 81 | 308 | CS | 243 cases & population control | Uncomplicated | A *vs* O (OR 1.06, 95% CI 0.72 – 1.55) B *vs* O (OR 0.81, 95% CI 0.55 – 1.19) AB *vs* O (OR 1.37, 95% CI 0.76 – 2.46) Non-O *vs* O (OR 0.91, 95% CI 0.67 – 1.24) | Microscopy |
| Singh et al. 1995 (India) | All ages | 2095 | PC | 14.7% | Uncomplicated | A *vs* O (OR 1.09,95% CI 0.78 – 1.55) B *vs* O (OR 1.39, 95% CI 1.01 – 1.91) AB *vs* O (OR 0.74, 95% CI 0.46 – 1.20) Non-O *vs* O (OR 1.16, 95% CI 0.87 – 1.56)  A *vs* O (RR 1.08, 95% CI 0.81 – 1.44) B *vs* O (RR 1.30, 95% CI 1.001 – 1.69) AB *vs* O (RR 0.78, 95% CI 0.51 – 1.18) Non-O *vs* O (RR 1.13, 95% CI 0.89 – 1.44) | Microscopy |
| Tadesse & Tadesse et al. 2013 (Ethiopia) | All ages | 398 | CS | 48.74 | Uncomplicated | A *vs* O (OR 0.59, 95% CI 0.35 – 0.98) B *vs* O (OR 0.46, 95% CI 0.27 – 0.81) Non-O *vs* O (OR 0.54, 95% CI 0.35 – 0.82) | Microscopy |
| Tekeste & Petros, 2010 (Ethiopia) | All ages | 400 | CS | 52.5% | Uncomplicated | A *vs* O (OR 0.98, 95% CI 0.58 – 1.67) B *vs* O (OR 0.81,95% CI 0.44 – 1.51) AB *vs* O (OR 0.59, 95% CI 0.27 – 1.32) Non-O *vs* O (OR 0.85, 95% CI 0.54 – 1.32) | Microscopy |
| Ukaga et al. 2007 (Nigeria) | Pregnant women | 586 | CS | 29.9% | Placental | A *vs* O (OR 0.72, 95% CI 0.46 – 1.13) B *vs* O (OR 0.68, 95% CI 0.39 – 1.18) AB *vs* O (OR 0.21, 95% CI 0.07 – 0.62) Non-O *vs* O (OR 0.61, 95% CI 0.42 – 0.89) | Microscopy |
| Uneke et al. 2006 (Nigeria) | 20-40 years | 325 | CS | 40.9% | Asymptomatic | A *vs* O (OR 0.96, 95% CI 0.42 – 2.22) B *vs* O (OR 1.05, 95% CI 0.41 – 2.69) Non-O *vs* O (OR 1.0, 95% CI 0.52 – 1.91) | Microscopy |
| Zerihun et al. 2011 (Ethiopia) | All ages | 269 | CS | 55.8% | Uncomplicated | A *vs* O (OR 2.52, 95% CI 1.28 – 4.97)  B *vs* O (OR 2.67, 95% CI 1.31 – 5.43)  AB *vs* O (OR 3.44, 95% CI 0.69 – 17.25)  Non-O *vs* O (OR 2.64, 95% CI 1.54 – 4.54)  Parasite density was lower among individuals with blood type O than those with blood type A or B | Microscopy |
| Bedu-Addo et al. 2014 (Ghana) | Pregnant women | 827 | CS | 62.3% | Placental | A *vs* O (OR 1.16, 95% CI 0.81 – 1.66)  B *vs* O (OR 1.62, 95% CI 1.09 – 2.39)  AB *vs* O (OR 1.77, 95% CI 0.80 – 3.91)  Non-O vs O (OR 1.38, 95% CI 1.04 –1.85) | Microscopy and PCR |
| Carvalho et al. 2010 (Brazil) | 18-65 | 240 | CC | 40.8% | Uncomplicated | A *vs* O (OR 1.05, 95% CI 0.55 – 1.99) B *vs* O (OR 0.66, 95% CI 0.29 – 1.49) AB *vs* O (OR 0.83, 95% CI 0.19 – 8.00) Non-O vs O (OR 0.88, 95% CI 0.52 – 1.49) | Microscopy and PCR |
| Kaisar et al. 2013 ((Indonesia) | 4-79 | 1,509 | CS | 14.5% (Based on PCR) | Asymptomatic/ uncomplicated | A *vs* O (OR 0.96, 95% CI 0.64 – 1.45) B *vs* O (OR 1.00: 95% CI 0.67 – 1.52) AB *vs* O (OR 1.55, 95% CI 0.79 – 3.04) Non-O *vs* O (OR 1.03, 95% CI 0.74 – 1.43) | Microscopy and PCR |
| Ojurongbe et al. 2011 (Nigeria) | Pregnant women | 179 | CS | 41.1% (based on PCR) | Asymptomatic | A *vs* O (OR 0.73, 95% CI 0·37 – 1.41) B *vs* O (OR 1.31, 95% CI 0.54 – 3.16) AB *vs* O (OR 3.03, 95% CI 0.55 – 16.64) Non-O *vs* O (OR 0.73, 95% CI 0.39 – 1.35) | Microscopy and PCR |
| Panda et al. 2012 (India) | 15-86 | 594 | CC | 83.2% | Uncomplicated | A *vs* O (OR 1.40, 95% CI 0.74 – 2.64) B *vs* O (OR 3.80, 95% CI 2.12 – 6.82) AB *vs* O (OR 1.16, 95% CI 0.51 – 2.64) Non-O *vs* O (OR 2.33, 95% CI 1.45, 3.75) | Microscopy and PCR |
| Panda et al. 2011 (India) | 15-80 | 527 | CC | 66.9% | Uncomplicated | A *vs* O (OR 0.79, 95% CI 0.41 – 1.52) B *vs* O (OR 0.68, 95% CI 0.37 – 1.25) AB *vs* O (OR 1.02, 95% CI 0.42 – 2.46) Non-O *vs* O (OR 0.77, 95% CI 0.47 – 1.28) | Microscopy and PCR *Not clear if results were based on PCR or Microscopy |
| Thakur & Verma, 1992 (India) | 5 to 65 | 258 | CS | 18.6% | Uncomplicated | A *vs* O (OR: 4.09, 95% CI: 1.27, 13.15) B *vs* O (OR: 2.42, 95% CI: 0.74, 7.91) AB *vs* O (OR: 1.10, 95% CI: 0.23, 5.26) Non-O *vs* O (OR: 2.65, 95% CI: 0.89, 7.88) | Microscopy  and ELISA |
| Rabha et al. 2012 (India) | All ages | 1,182 | CS | 16.2% | Asymptomatic/ Uncomplicated | Each blood type has equal susceptibility to *P. falciparum* infection | Microscopy and RDT |
| Nwauche et al. 2011 (Nigeria) | 16-60 | 246 | CS | 25.2% | Asymptomatic/ Uncomplicated | A *vs* O (OR 0.88 95% CI 0·41 – 1.89) B *vs* O (OR 0.36, 95% CI 0.10 – 1.26) Non-O *vs* O (OR 0.67, 95% CI 0.34 – 1.33) | PCR |
| Adam et al. 2009 (Sudan) | Pregnant women (25.5±6.0) | 236 | CS | 19.5%  (based on histology) | Placental | Non-O *vs* O (OR 1.25, 95% CI 0.91 – 2.5) | Microscopy and Histology |
| Adam et al. 2007 (Sudan) | Pregnant women (25.5±6.4) | 293 | CS | 32.0% (based on histology) | Placental | A *vs* O (OR 0.80, 95% CI: 0.44 –1.44) B *vs* O (OR 0.59, 95% CI: 0.30 – 1.17) AB *vs* O (OR 0.57, 95% CI, 0.21 – 1.55) Non-O *vs* O (OR 0.69, 95% CI: 0.21 – 1.12) Non-O *vs* O for passive infection (OR 0.53, 95 CI 0.31 – 0.90) | Microscopy and Histology |
| Alim et al. 2015 (Sudan) | Pregnant women | 126 | CS | 19.8% | Placental | Non-O *vs* O (aOR 1.11, 95% CI 0.41 – 3.33) | Microscopy and Histology |
| Loscertales & Brabin, 2006 (Gambia) | Mothers | 198 | CS | 59% | Placental | A *vs* O (OR 1.35, 95% CI 0.63 – 2.91) B *vs* O (OR 1.17, 95% CI 0.59 – 2.29) AB *vs* O (OR 1.58, 95% CI 0.28 – 9.01) Non-O *vs* O (OR 1.26, 95% CI 0.71 – 2.22) | Microscopy and Histology |
| Adegnika et al. 2011 (Gabon) | Pregnant women (25.5±6.4) | 378 | CS | 37.4% (based on histology) | Placental | A *vs* O (OR 1.11, 95% CI 0.39 – 3.19) B *vs* O (OR 2.38, 95% CI 0.91 – 6.23) AB *vs* O (OR 2, 95% CI 0.23 – 17.13) Non-O vs O (OR 1.64, 95% CI 0.75 – 3.59) | Histology |
| Senga et al. 2007 (Malawi) | 15-49 | 647 | CS | 36.3% | Placental | A *vs* O (OR 0.75, 95% CI 0.48 – 1.17) B *vs* O (OR 1.22, 95% CI 0.83 – 1.79) AB *vs* O (OR 3.68, 95% CI 1.39 – 9.74) Non-O *vs* O (OR 1.09, 95% CI 0.79 – 1.51) | Histology |

aOR: adjusted odds ratio; aRR: adjusted relative risk; CC: Case Control; CS: Cross-sectional; ELISA: enzyme-linked immunosorbent assay; OR: Odds Ratio; PCR: Polymerase Chain Reaction; RR: Relative risk; PC: Prospective control
