# Supplementary material for: Effect of ABO blood group on asymptomatic, uncomplicated and placental Plasmodium falciparum infection: systematic review and meta-analysis
Source: BMC Infect Dis. 2019 Jan 25;19:86. doi: 10.1186/s12879-019-3730-z (PMC6346527; doi:10.1186/s12879-019-3730-z)
Supplement: Supplementary file 4 — Figure S1. Funnel plot. Odds ratio against standard error of odds ratio for studies, which compared the odds of uncomplicated Plasmodium falciparum infection vs Plasmodium uninfected among individuals with blood group A vs O, B vs O, AB vs O and Non-O vs O. (DOCX 279 kb) [file 12879_2019_3730_MOESM4_ESM.docx]

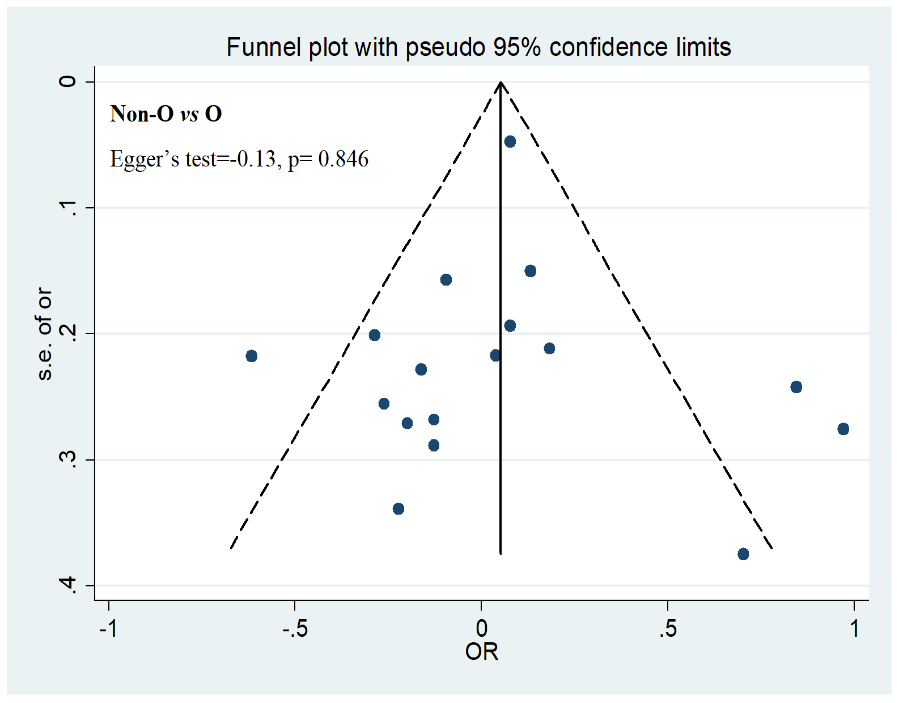

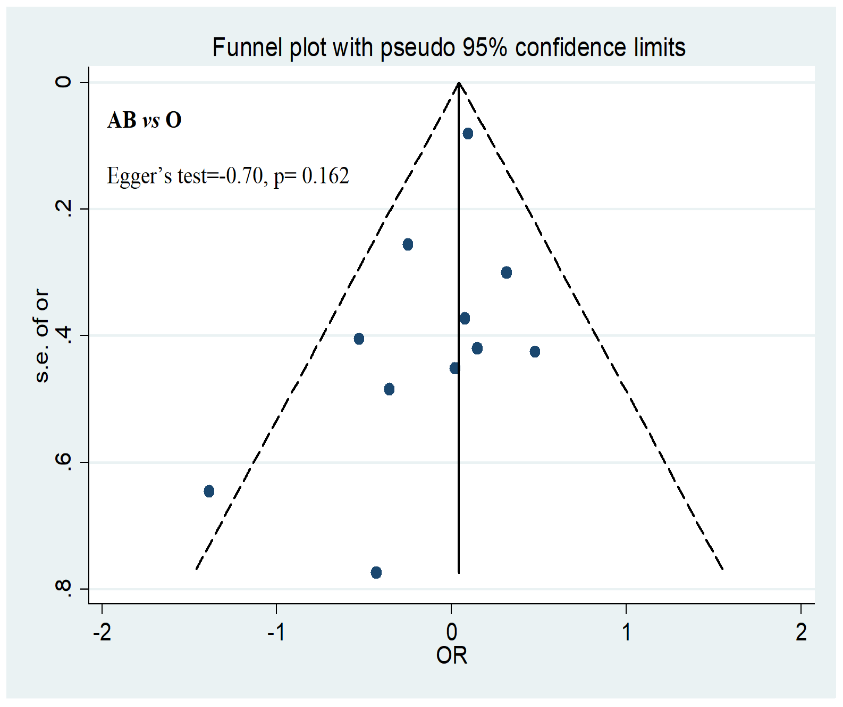

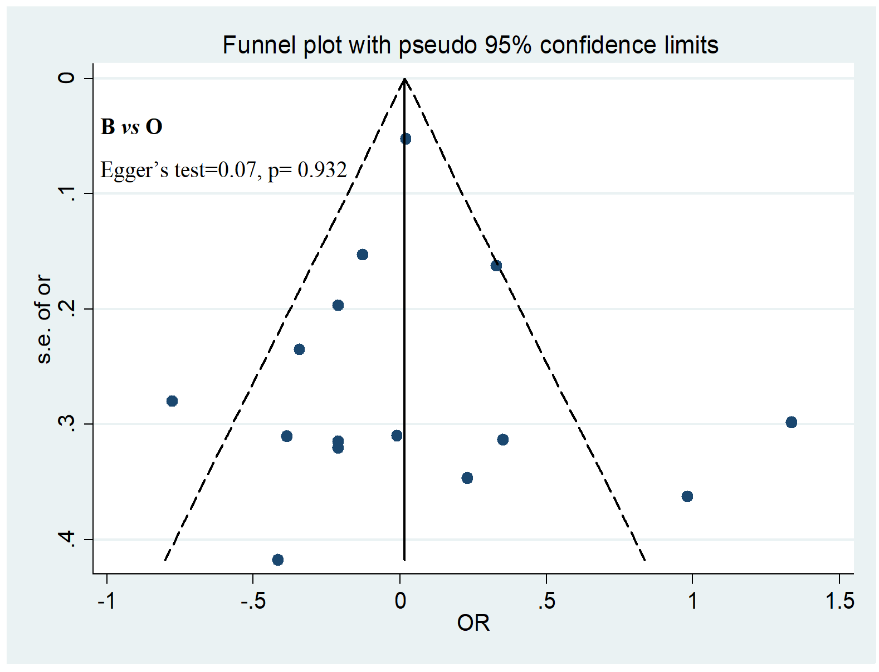

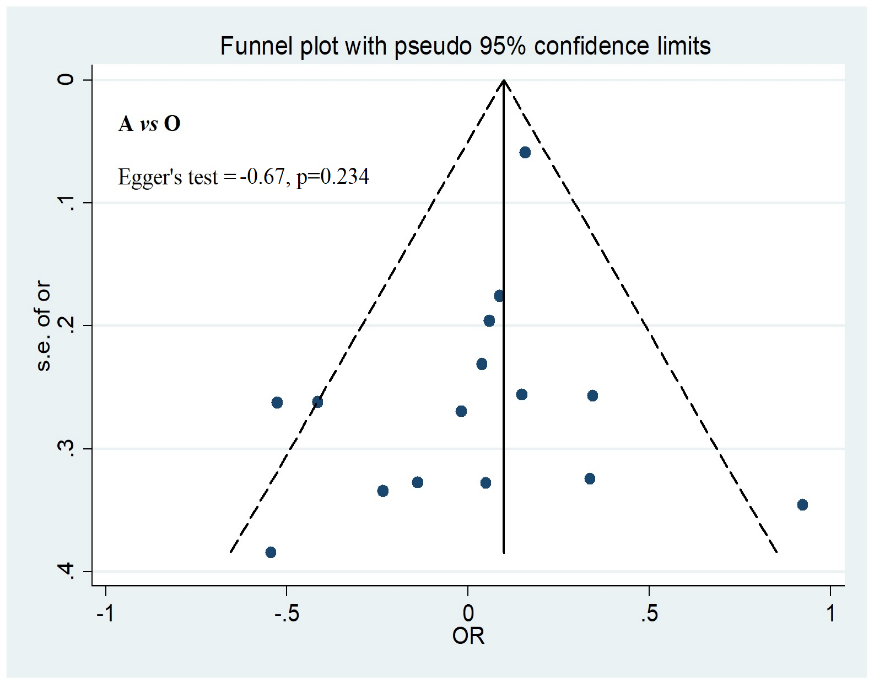


Additional file 4: Fig S1. Funnel plot. Odds ratio against standard error of odds ratio for studies, which compared the odds of uncomplicated *Plasmodium falciparum* infection *vs* *Plasmodium* uninfected among individuals with blood group A *vs* O, B *vs* O, AB *vs* O and Non-O *vs* O
