# Supplementary material for: Effect of ABO blood group on asymptomatic, uncomplicated and placental Plasmodium falciparum infection: systematic review and meta-analysis
Source: BMC Infect Dis. 2019 Jan 25;19:86. doi: 10.1186/s12879-019-3730-z (PMC6346527; doi:10.1186/s12879-019-3730-z)
Supplement: Supplementary file 5 — Figure S2. Funnel plot. Odds ratio against standard error of odds ratio for studies, which compared the odds of asymptomatic Plasmodium falciparum infection vs Plasmodium uninfected among individuals with blood group A vs O, B vs O, AB vs O and Non-O vs O. (DOCX 244 kb) [file 12879_2019_3730_MOESM5_ESM.docx]

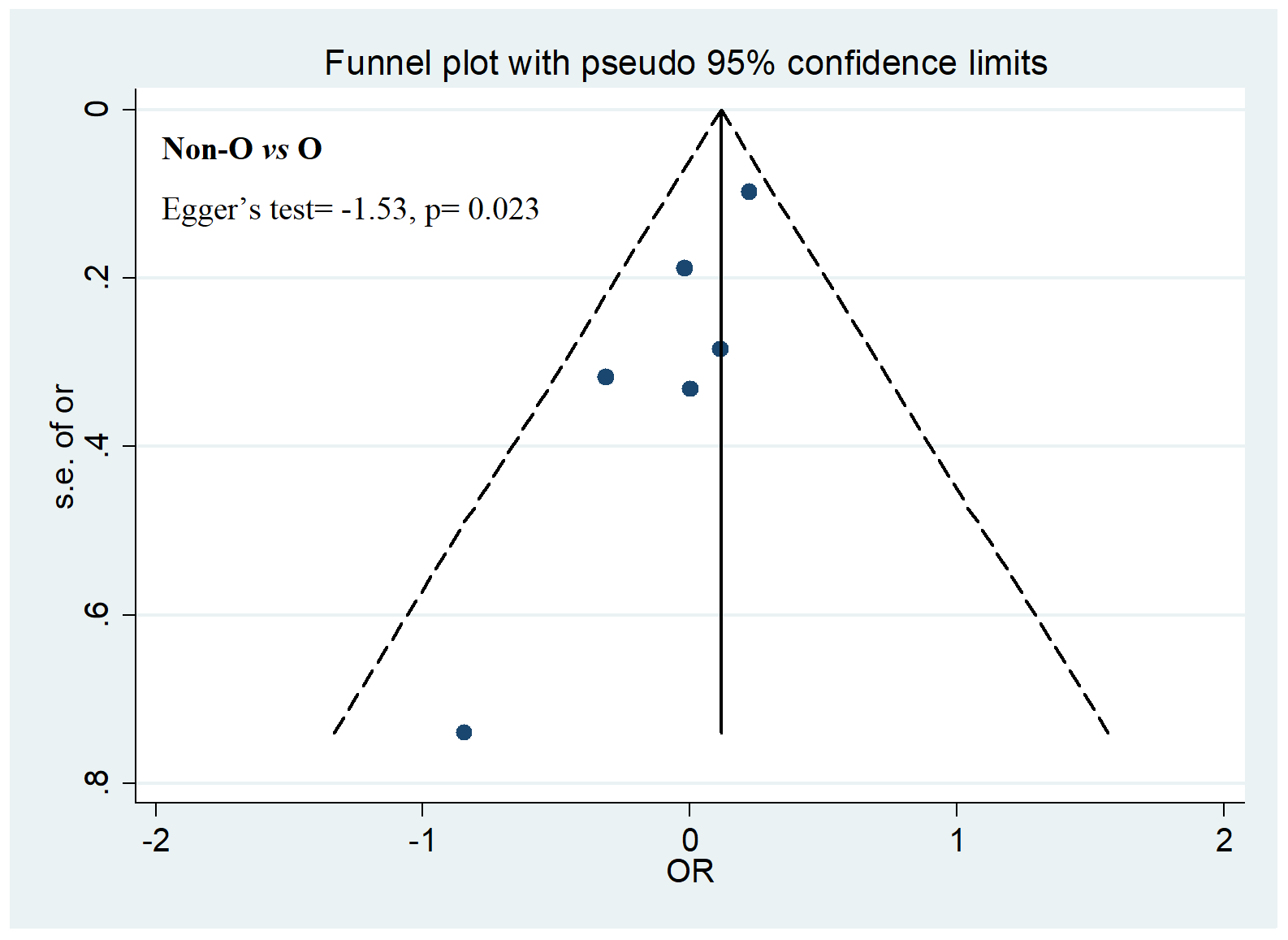

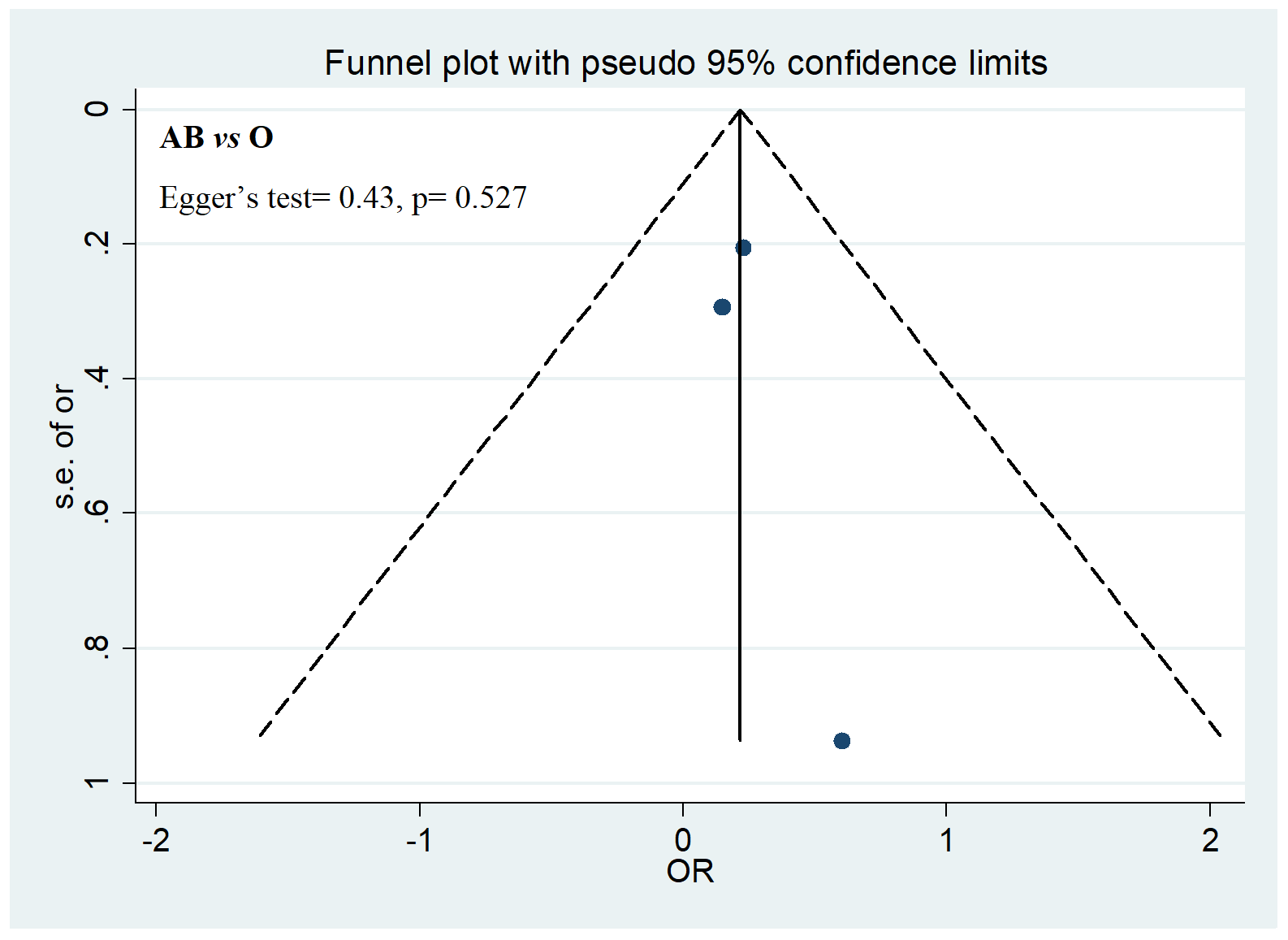

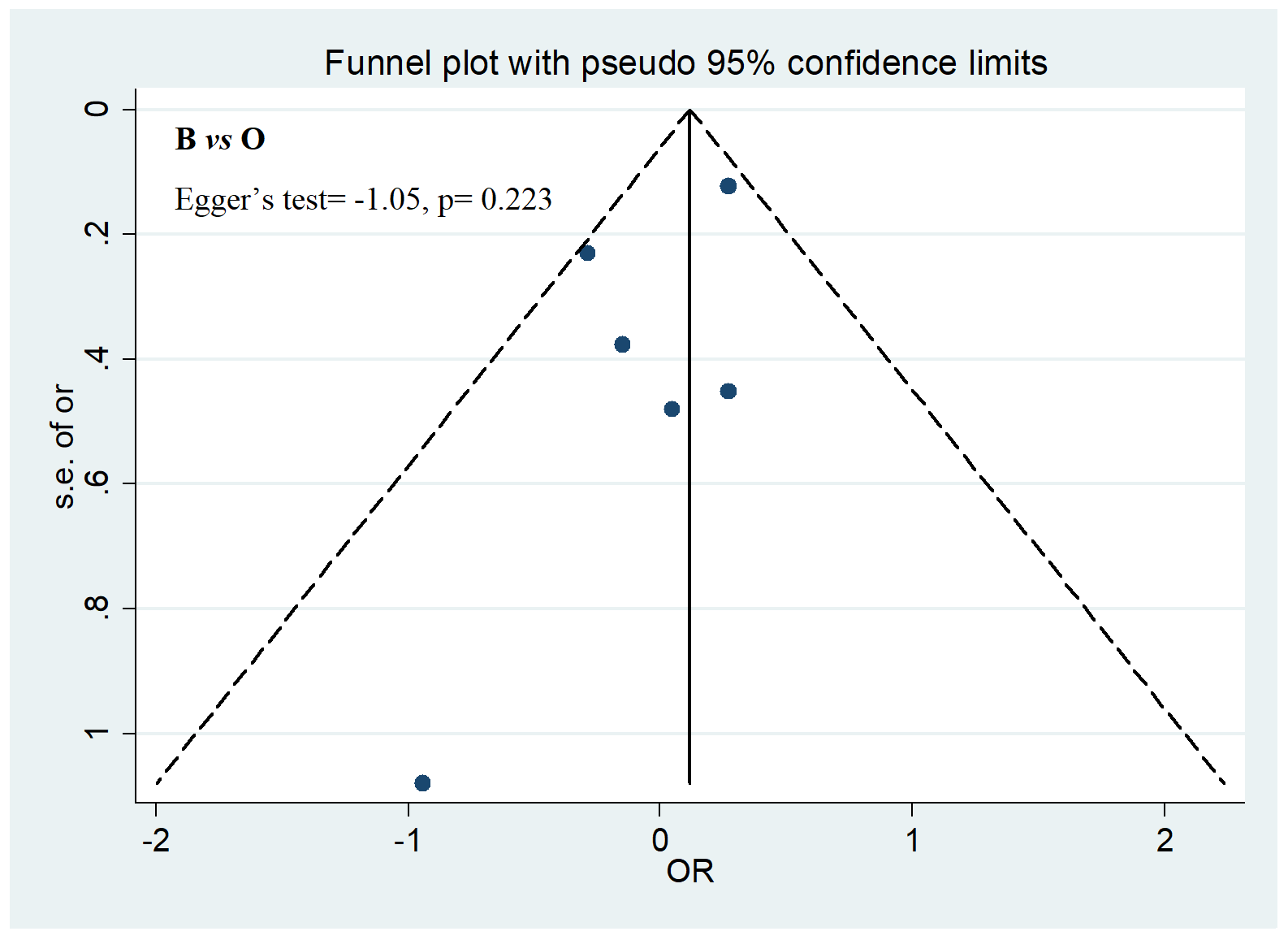

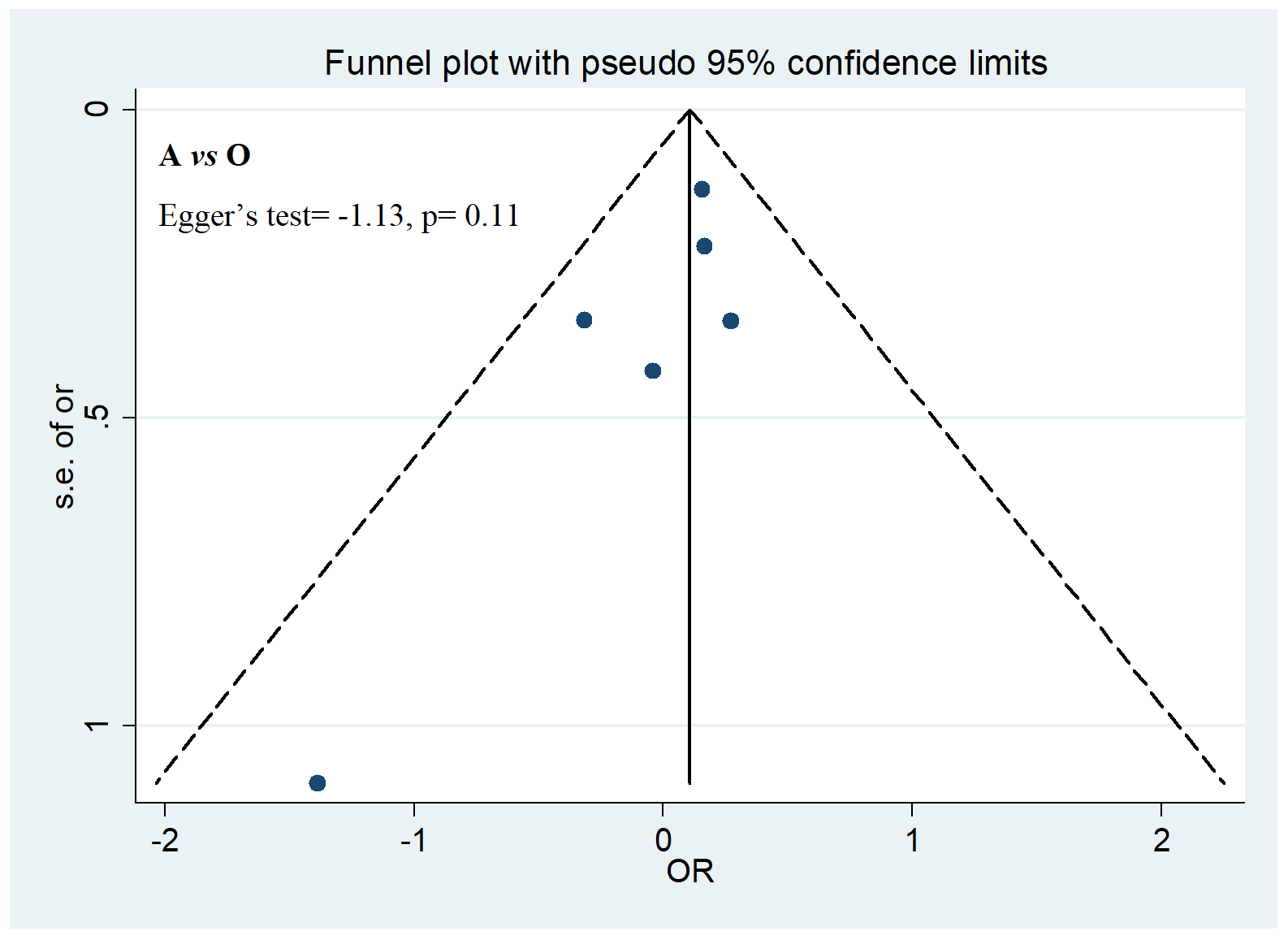


Additional file 5: Fig S2. Funnel plots. Odds ratio against standard error of odds ratio for studies, which compared the odds of asymptomatic *Plasmodium falciparum* infection *vs* *Plasmodium* uninfected among individuals with blood group A *vs* O, B *vs* O, AB *vs* O and Non-O *vs* O
