# Supplementary material for: Effect of ABO blood group on asymptomatic, uncomplicated and placental Plasmodium falciparum infection: systematic review and meta-analysis
Source: BMC Infect Dis. 2019 Jan 25;19:86. doi: 10.1186/s12879-019-3730-z (PMC6346527; doi:10.1186/s12879-019-3730-z)
Supplement: Supplementary file 7 — Table S4. Sources of heterogeneity assessment based on meta-regression analyses. (DOCX 16 kb) [file 12879_2019_3730_MOESM7_ESM.docx]

Additional file 7: Table S4. Sources of heterogeneity assessment based on meta-regression analyses.

| Type of malaria compared | Blood group Comparison | Factors | Meta-regression  Coefficient | LCI | UCI | p-value |
| --- | --- | --- | --- | --- | --- | --- |
| Uncomplicated *vs* uninfected | A *vs* O | Sample size | 1.00 | 0.99 | 1.00 | 0.705 |
|  |  | Study region | 1.02 | 0.64 | 1.62 | 0.925 |
|  |  | Study design | 0.94 | 0.59 | 1.51 | 0.790 |
|  |  | Age | 1.11 | 0.63 | 1.96 | 0.680 |
|  | B *vs* O | Sample size | 1.00 | 0.99 | 1.00 | 0.879 |
|  |  | Study region | 1.07 | 0.42 | 2.71 | 0.881 |
|  |  | Study design | 0.76 | 0.29 | 2.03 | 0.551 |
|  |  | Age | 0.78 | 0.25 | 2.44 | 0.635 |
|  | AB *vs* O | Sample size | 2.19 | 0.99 | 1.00 | 0.794 |
|  |  | Study region | 0.93 | 0.47 | 1.84 | 0.814 |
|  |  | Study design | 1.09 | 0.39 | 2.97 | 0.569 |
|  |  | Age | 0.81 | 0 .34 | 1.932 | 0.845 |
|  | Non-O *vs* O | Sample size | 2.00 | 0.99 | 1.00 | 0.945 |
|  |  | Study region | 0.92 | 0.54 | 1.57 | 0.749 |
|  |  | Study design | 0.89 | 0.49 | 1.61 | 0.669 |
|  |  | Age | 0.86 | 0.45 | 1.66 | 0.635 |
| Asymptomatic *vs* uninfected | A *vs* O | Sample size | 1.00 | 0.99 | 1.00 | 0.561 |
|  |  | Study region | 2.49 | 0.02 | 317.26 | 0.503 |
|  |  | Age | 2.16 | 0.02 | 267.99 | 0.562 |
|  | B *vs* O | Sample size | 1.00 | 0.99 | 1.00 | 0.442 |
|  |  | Study region | 1.39 | 0.01 | 182.90 | 0.797 |
|  |  | Age | 1.99 | 0.02 | 259.61 | 0.603 |
|  | AB *vs* O | Insufficient observation | ----- | ------ | ------- |  |
|  | Non-O *vs* O | Sample size | 1.00 | 0.99 | 1.00 | 0.286 |
|  |  | Study region | 1.68 | 0.06 | 44.32 | 0.565 |
|  |  | Age | 1.76 | 0.07 | 44.85 | 0.530 |

Study region (Africa *vs* Asia), Study design (cross-sectional *vs* Case control *vs* Cohort), Age (Children *vs* Adult *vs* All ages)

Method used for the diagnosis of *Plasmodium* infection was microscope except for two studies

The design of all studies that compared the odds of asymptomatic *vs* uninfected among individuals with different blood groups was cross-sectional
